# A Case of VF Misclassification Corrected by VF Therapy Assurance: Implications for ICD Programming

**DOI:** 10.1002/joa3.70265

**Published:** 2026-01-07

**Authors:** Naoki Matsumoto, Kenji Shimeno, Masanori Matsuo, Yukio Abe, Daiju Fukuda

**Affiliations:** ^1^ Department of Cardiology Osaka City General Hospital Osaka Japan; ^2^ Department of Cardiovascular Medicine Osaka Metropolitan University Graduate School of Medicine Osaka Japan

**Keywords:** implantable cardioverter defibrillator, ventricular fibrillation, ventricular fibrillation therapy assurance

## Abstract

Implantable cardioverter‐defibrillator undersensing may delay therapy in ventricular fibrillation with low‐amplitude signals. Ventricular fibrillation therapy assurance (VFTA) detected persistent VF after initial shock failure, enabling timely shock delivery and successful resuscitation. VFTA may help optimize device programming by preventing misclassification and treatment delay in life‐threatening arrhythmias.

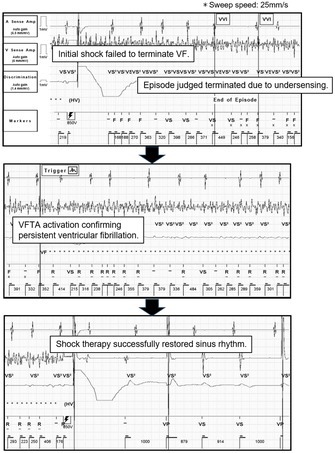

## 1 Case Presentation

**FIGURE 1:**
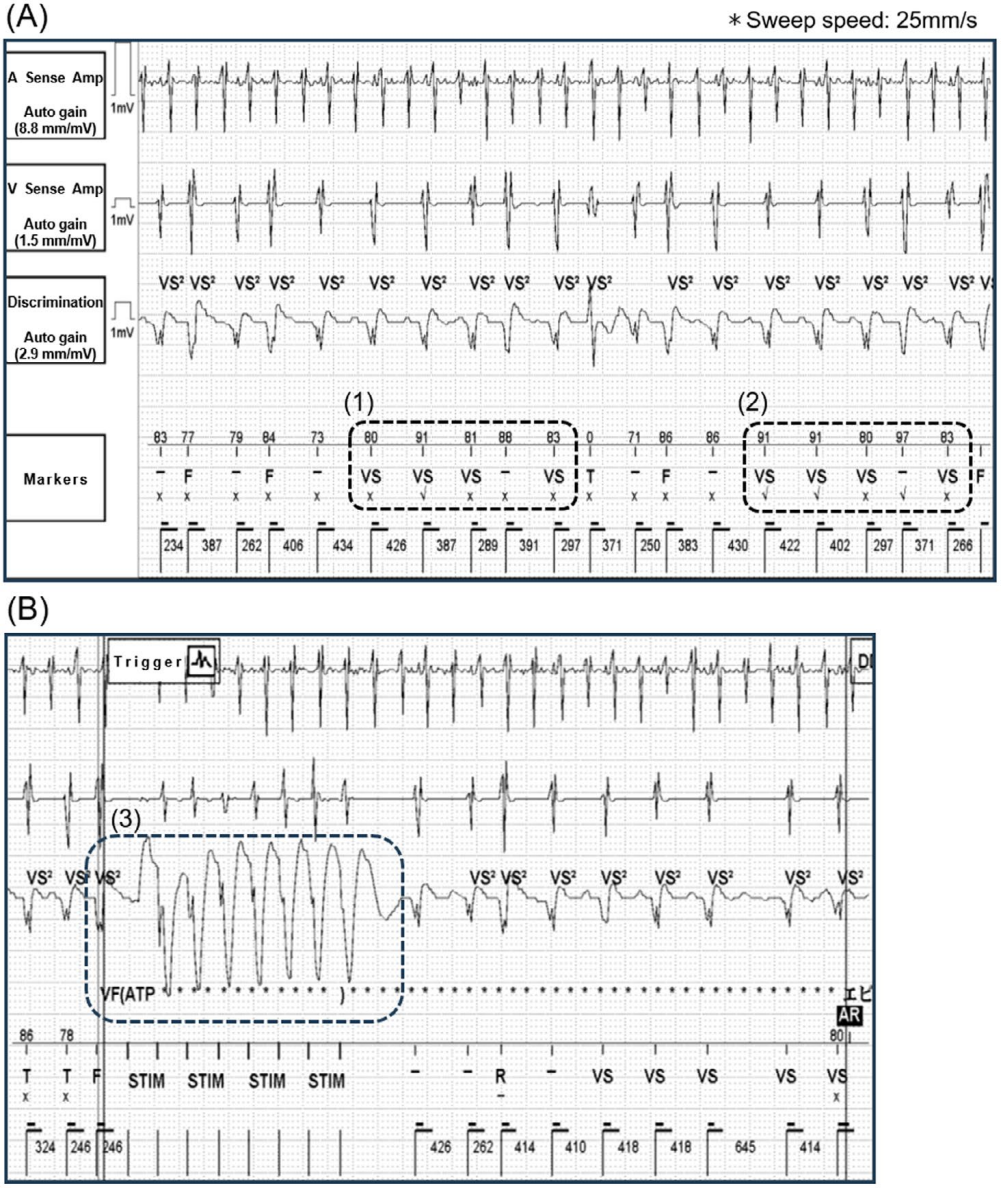
(A) Electrogram of atrial fibrillation (AF). The VS markers within the dotted line indicate 4 beats (A‐(1) and A‐(2)), which do not fulfill the criteria for tachycardia episode termination. (B) Anti‐tachycardia pacing (ATP) therapy was delivered due to misclassification of AF as ventricular fibrillation (B‐(3)). AR, Accelerated Rhythm; ATP, anti‐tachycardia pacing; F, ventricular interval within ventricular fibrillation zone; R, reconfirmed interval; STIM, stimulus of anti‐tachycardia pacing; T, ventricular interval within ventricular tachycardia zone; VS, Ventricular Sensed event; VS2, low‐amplitude R‐wave signal detected by the coil‐to‐can sensing channel (discrimination channel).

**FIGURE 2:**
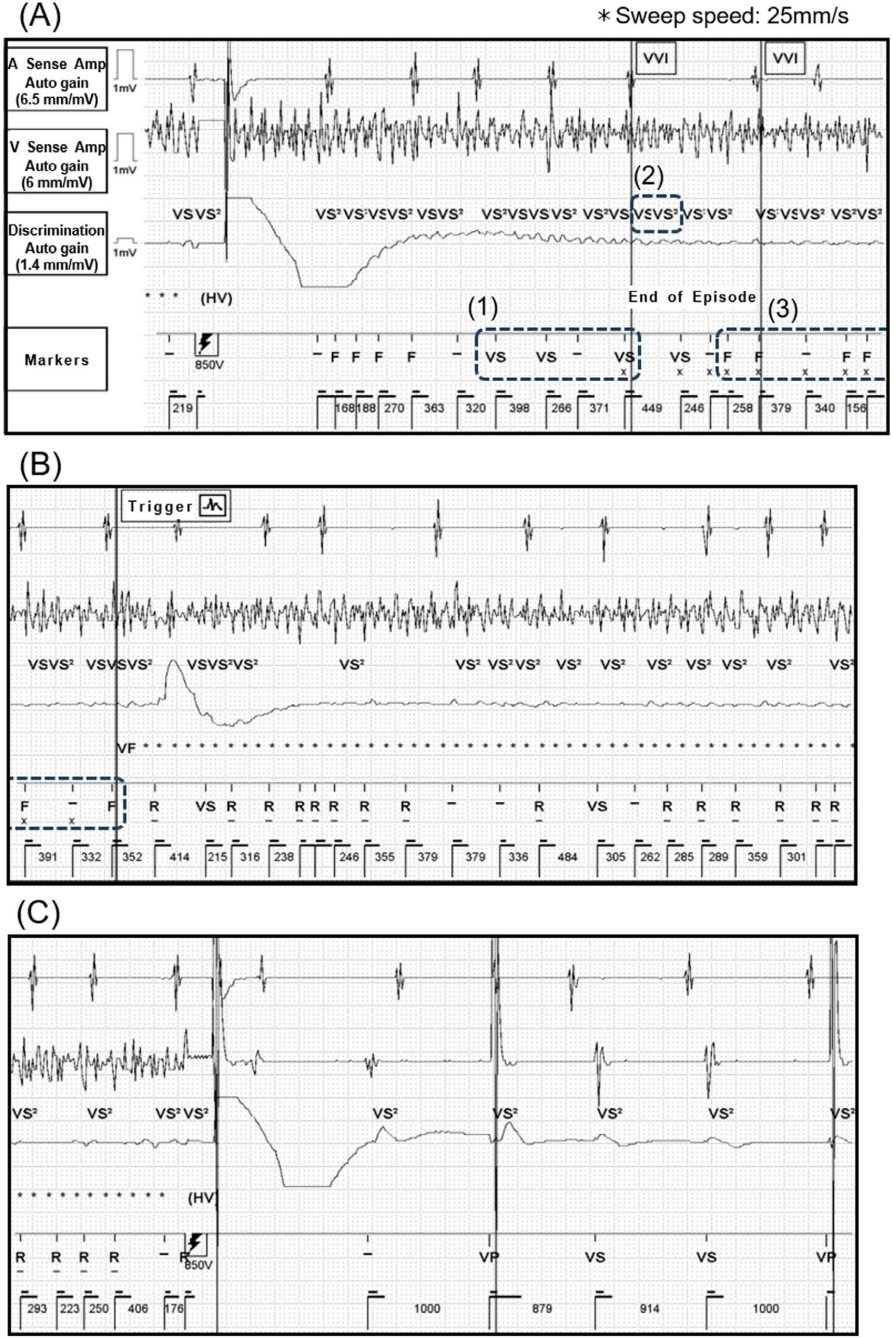
(A) A shock was delivered to treat ventricular fibrillation (VF); however, VF was not terminated successfully. Subsequently, undersensing occurred, resulting in the detection of only 3 consecutive VS markers, causing the episode to be incorrectly classified as terminated (A‐(1)). Shortly thereafter, 2 consecutive VS2 markers were detected in the discrimination channel, triggering ventricular fibrillation therapy assurance (VFTA) (A‐(2)). After VFTA activation, 6 F markers were detected, confirming VF (A‐(3) and B). (B) Charging was initiated to prepare for shock delivery. (C) The shock therapy successfully restored sinus rhythm. HV, high‐voltage shock therapy; VVI, ventricular pacing, ventricular sensing, inhibited response; other abbreviations are as defined above.

A 58‐year‐old female with a family history of sudden cardiac death experienced ventricular fibrillation (VF) during malignant lymphoma treatment. She was diagnosed with idiopathic VF and underwent implantation of an implantable cardioverter‐defibrillator (ICD) (Gallant DR, Abbott, Sylmar, CA) for secondary prevention, along with an atrial lead (Tendril STS 2088TC, Abbott, Sylmar, CA) and a ventricular lead (Durata 7122Q, Abbott, Sylmar, CA). The right ventricular (RV) amplitude at implantation was 6.5 mV and remained stable, ranging from 5–7 mV thereafter (Supplemental Figure S1A). The tachycardia detection and therapy settings were as follows: The VF zone was set at ≥ 222 bpm with a Number of Intervals to Detect (NID) of 30, while the ventricular tachycardia (VT) zone was set at ≥ 171 bpm with an NID of 30 as a monitor zone. Termination criteria were set at 5 consecutive Ventricular Sensed (VS) markers, and post‐shock redetection was set at 6 VF (F) markers, with the sensitivity for VF detection at 0.5 mV. She later experienced atrial fibrillation (AF), which was misclassified as VF (Figure 1A). During shock charging, anti‐tachycardia pacing (ATP) was delivered (Figure 1B), which incidentally terminated the episode. As ATP was applied before accumulating 5 VS markers (Figure 1A), the event would have continued to be classified as VF if the termination criterion had remained unchanged. To prevent inappropriate future therapy, the termination criterion was reduced to 3 VS markers. No other therapy settings were modified. She subsequently underwent catheter ablation for AF. Despite the procedure, AF control remained poor, and there was a trend toward worsening heart failure (Supplemental Figures S1B and S2). Under these circumstances, she experienced VF. An appropriate ICD shock for VF was delivered but failed to terminate the arrhythmia. During VF, a gradual reduction in signal amplitude was observed, which further decreased after the unsuccessful first shock (Supplemental Figure S3), leading to misclassification of the ongoing VF episode as terminated based on 3 consecutive VS markers. However, ventricular fibrillation therapy assurance (VFTA) detected sustained VF and triggered an additional shock that successfully restored sinus rhythm (Figure 2). Afterward, a β‐blocker was initiated, resulting in satisfactory control of both AF and VF. Given the importance of preventing undersensing observed during this VF episode, the VF detection sensitivity was changed from 0.5 mV to 0.3 mV, and the termination criterion was returned to 5 consecutive VS markers. No other therapy parameters were modified, and the VFTA function was kept activated. She continues to be followed as an outpatient, with no recurrence of AF or VF observed to date.

## 2 Discussion

**FIGURE 3:**
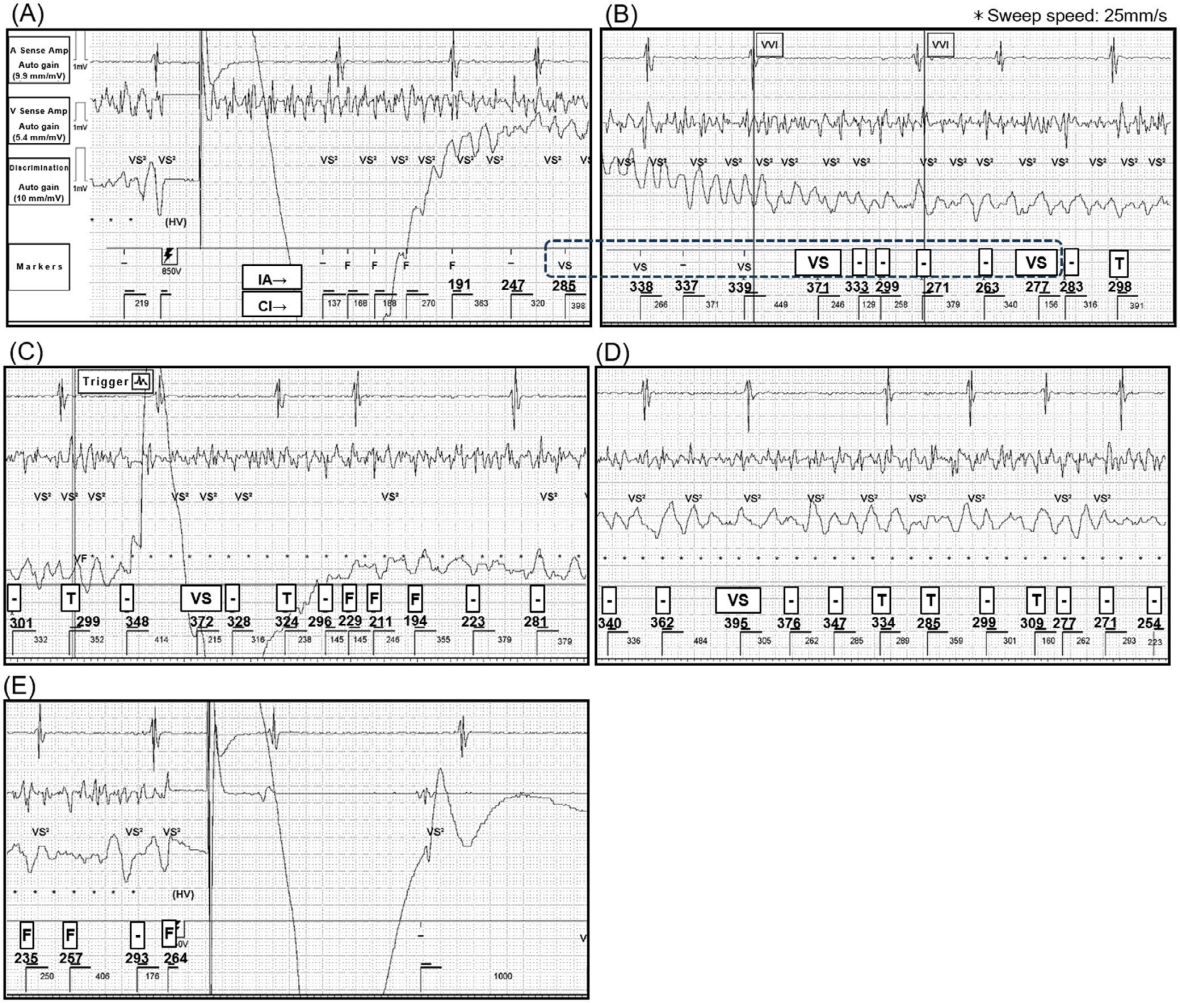
(A–E) Simulated electrogram illustrating the scenario in which ventricular fibrillation therapy assurance (VFTA) was turned off. Markers were recalculated using the binning method, incorporating Interval Average (IA) and Current Interval (CI). (A and B) Ventricular fibrillation (VF) persisted even after shock therapy. If the VFTA had been off, the VS markers (within the dotted line) would have reached 5, meeting the tachycardia episode termination criteria. (C–E) By the time the second shock was delivered with the VFTA function on, only 6 F markers had been detected in this simulation, which likely failed to meet the NID30 requirement, resulting in failure to detect VF. IA (Mean of CI and the preceding three beats) is displayed in the upper row. CI (Mean of the preceding two beats) is displayed in the lower row. CI, Current Interval; IA, Interval Average; Other abbreviations are as defined above.

VFTA is a feature available in Gallant (Abbott, Sylmar, CA) and Entrant ICDs (Abbott, Sylmar, CA), designed to address undersensing in polymorphic VT and VF. As VF progresses, RV amplitude may decrease [1], causing signal fluctuations that lead to misclassification. VFTA utilizes a discrimination channel to monitor potentials between the device and the RV coil. When the amplitude falls within 0.3–0.6 mV, VS2 markers are assigned. VFTA is triggered when continuous VS2 markers (Low‐Amplitude Counter) or an absence of VS2 markers for 2 s (Pause Counter) is detected. When VFTA is activated, the VF detection rate is adjusted to the slowest treatment cycle + 100 ms, switching to a mode that only administers shock therapy. VF detection is performed using an NID of 6 intervals, while episode termination requires 7 sinus intervals, thereby enhancing sensitivity to critical arrhythmias [2]. Current guidelines advocate for higher‐rate ICD programming, prolonged detection intervals, and supraventricular arrhythmia discrimination to prevent inappropriate therapy [3]. However, these strategies may delay life‐saving treatment. Delayed ICD therapy has been associated with signal amplitude reduction and poor post‐cardiac arrest outcomes [4]. In this case, although ICD programming with a high‐rate cutoff and prolonged detection intervals aimed at reducing inappropriate therapy may have contributed to undersensing, the time from VF onset to the first shock was only 24 s, indicating that VF was not sustained for a prolonged period. During this period, the patient exhibited worsening heart failure in the setting of AF with rapid ventricular response, accompanied by reduced RV amplitudes (Supplemental Figures S1A and S2), possibly due to myocardial edema. We hypothesize that the underlying heart failure may have contributed to undersensing despite the VF episode lasting less than 30 s. A retrospective analysis was conducted under the assumption that VFTA was disabled during this episode (Figure 3). It was found that tachycardia termination would have been misjudged regardless of whether the termination criterion was set at 3 or 5 consecutive VS markers. Furthermore, due to the low amplitude, the subsequent accumulation of F markers was insufficient, making it highly unlikely to reach the required threshold of 30 F markers for a new VF detection. Therefore, it is likely that she would not have been successfully resuscitated without VFTA. Okubo et al. previously reported a case in which VF occurring after ATP for VT was not detected despite a highly sensitive detection setting (NID = 12), due to low signal amplitude, whereas appropriate shock delivery was achieved through activation of VFTA. They concluded that VF would not have been detected using the binning‐based algorithm alone [5]. Although the present case differs from their report in that the gradually decreasing signal amplitude led to misclassification of VF as sinus rhythm recovery after the first shock, the underlying mechanism in both cases was a progressive reduction in signal amplitude during VF, which underscores the limitation of the binning‐based detection algorithm and suggests that VFTA may compensate for this limitation.

Additionally, in this case, AF was misclassified as VF. Supraventricular tachyarrhythmias are the primary causes of inappropriate therapy in transvenous ICDs [6], and even inappropriate therapy doubles the mortality risk [7]. To address this, the termination criterion was adjusted to 3 VS markers. Alternative approaches, such as modifying the VT zone or VF threshold, may reduce inappropriate therapy but risk delaying VF treatment and increasing the likelihood of undersensing. In this case, the adjustment of the termination criterion was ineffective, as the episode was incorrectly considered terminated despite ongoing VF following the shock. Clinicians often face the challenge of balancing timely interventions for life‐threatening arrhythmias while preventing inappropriate treatments. However, VFTA has the potential to reduce failures to detect lethal arrhythmias requiring treatment, even when programmed with a focus on minimizing inappropriate activations. VFTA can be a valuable feature that provides various setting options when considering the optimal configuration for tachycardia therapy.

## Funding

This study was not supported by any funding.

## Ethics Statement

Informed consent was obtained from the patient to publish the case report.

## Consent

Patient consent was obtained for this report.

## Conflicts of Interest

The authors declare no conflicts of interest.

## Supporting information


**Supplemental Figure 1** (A) The graph illustrates the longitudinal trend of right ventricular (RV) amplitude with bipolar polarity. For most of the observation period, the RV amplitude remained stable at 5–7 mV. However, during the phase of sustained atrial fibrillation (AF) preceding the onset of ventricular fibrillation, concomitant with a trend toward worsening heart failure, the amplitude decreased to approximately 4 mV. (B) The graph illustrates the longitudinal trend of atrial tachycardia and AF burden. Despite undergoing catheter ablation, AF control remained suboptimal; however, the introduction of a β‐blocker subsequently resulted in improved control. AF, atrial fibrillation; AT, atrial tachycardia; VF, ventricular fibrillation.

**Supplemental Figure 2** Chest radiographs obtained after implantable cardioverter‐defibrillator implantation (A) and during a period of high atrial fibrillation burden (B). (B) Compared with (A), the chest radiograph demonstrates worsening pulmonary congestion, and the cardiothoracic ratio increased from 45% to 54%. AF, atrial fibrillation; ICD, implantable cardioverter‐defibrillator.

**Supplemental Figure 3** The intracardiac electrogram from ventricular fibrillation detection to the delivery of the first shock is shown. When comparing the amplitude of the near‐field right ventricular signal in panel (A‐1) with panel (B‐2), it is evident that the signal amplitude in panel (B‐2) is markedly reduced. This progressive reduction in amplitude resulted in undersensing after the shock, which subsequently triggered ventricular fibrillation therapy assurance activation. F, ventricular interval within ventricular fibrillation zone; HV, high‐voltage shock therapy; R, reconfirmed interval; STIM, stimulus of anti‐tachycardia pacing; T, ventricular interval within ventricular tachycardia zone; VS, Ventricular Sensed event; VS2, low‐amplitude R‐wave signal detected by the coil‐to‐can sensing channel (discrimination channel); VVI, ventricular pacing, ventricular sensing, inhibited response.

## Data Availability

The data underlying the results are available in this article.
