# Supplementary figures and images for: A Case of VF Misclassification Corrected by VF Therapy Assurance: Implications for ICD Programming
Source: J Arrhythm. 2026 Jan 7;42(1):e70265. doi: 10.1002/joa3.70265 (PMC12779415; doi:10.1002/joa3.70265)

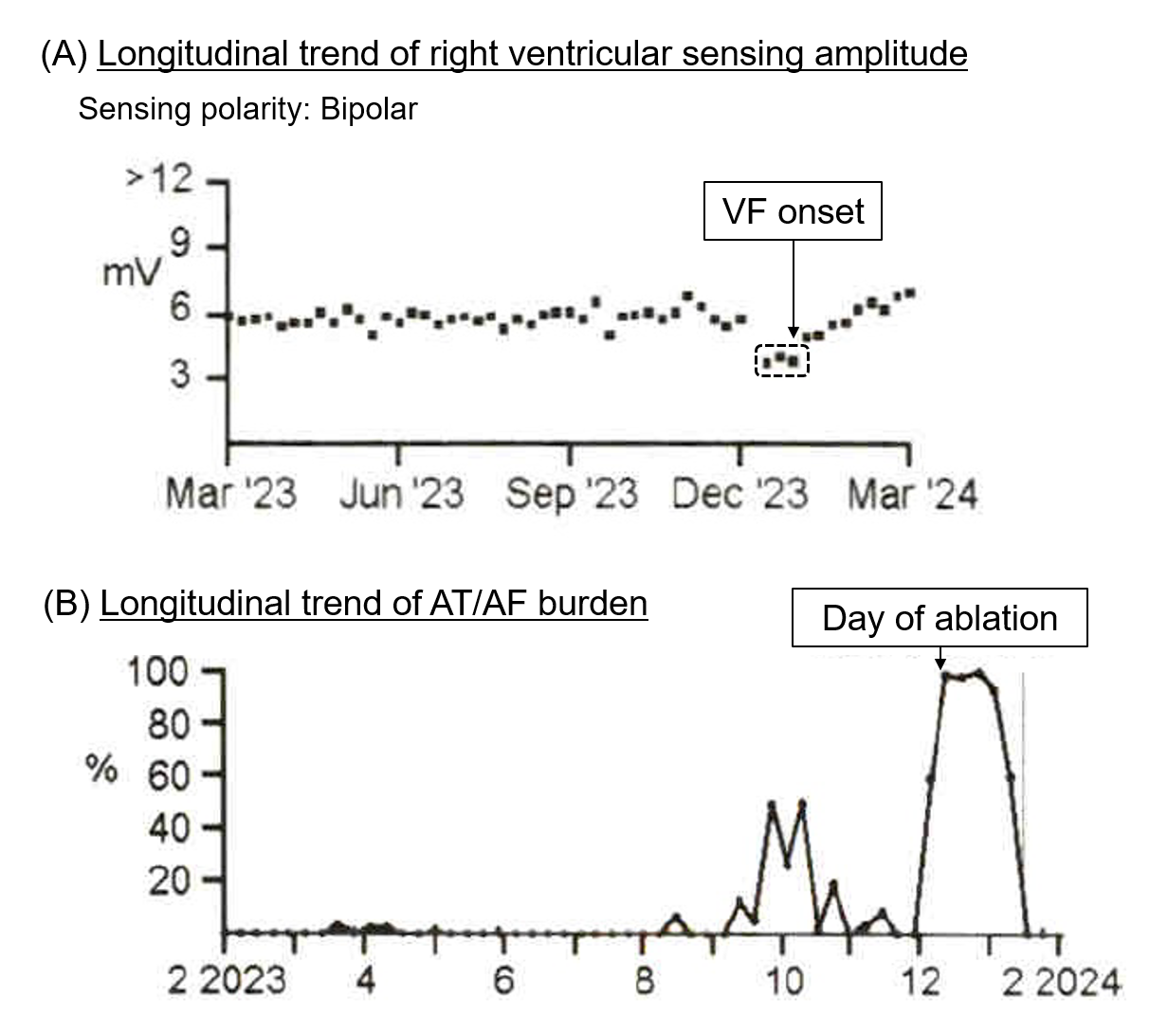

Supplement: Supplementary file 1 — Supplemental Figure 1 (A) The graph illustrates the longitudinal trend of right ventricular (RV) amplitude with bipolar polarity. For most of the observation period, the RV amplitude remained stable at 5–7 mV. However, during the phase of sustained atrial fibrillation (AF) preceding the onset of ventricular fibrillation, concomitant with a trend toward worsening heart failure, the amplitude decreased to approximately 4 mV. (B) The graph illustrates the longitudinal trend of atrial tachycardia and AF burden. Despite undergoing catheter ablation, AF control remained suboptimal; however, the introduction of a β‐blocker subsequently resulted in improved control. AF, atrial fibrillation; AT, atrial tachycardia; VF, ventricular fibrillation. [file JOA3-42-e70265-s001.tif]

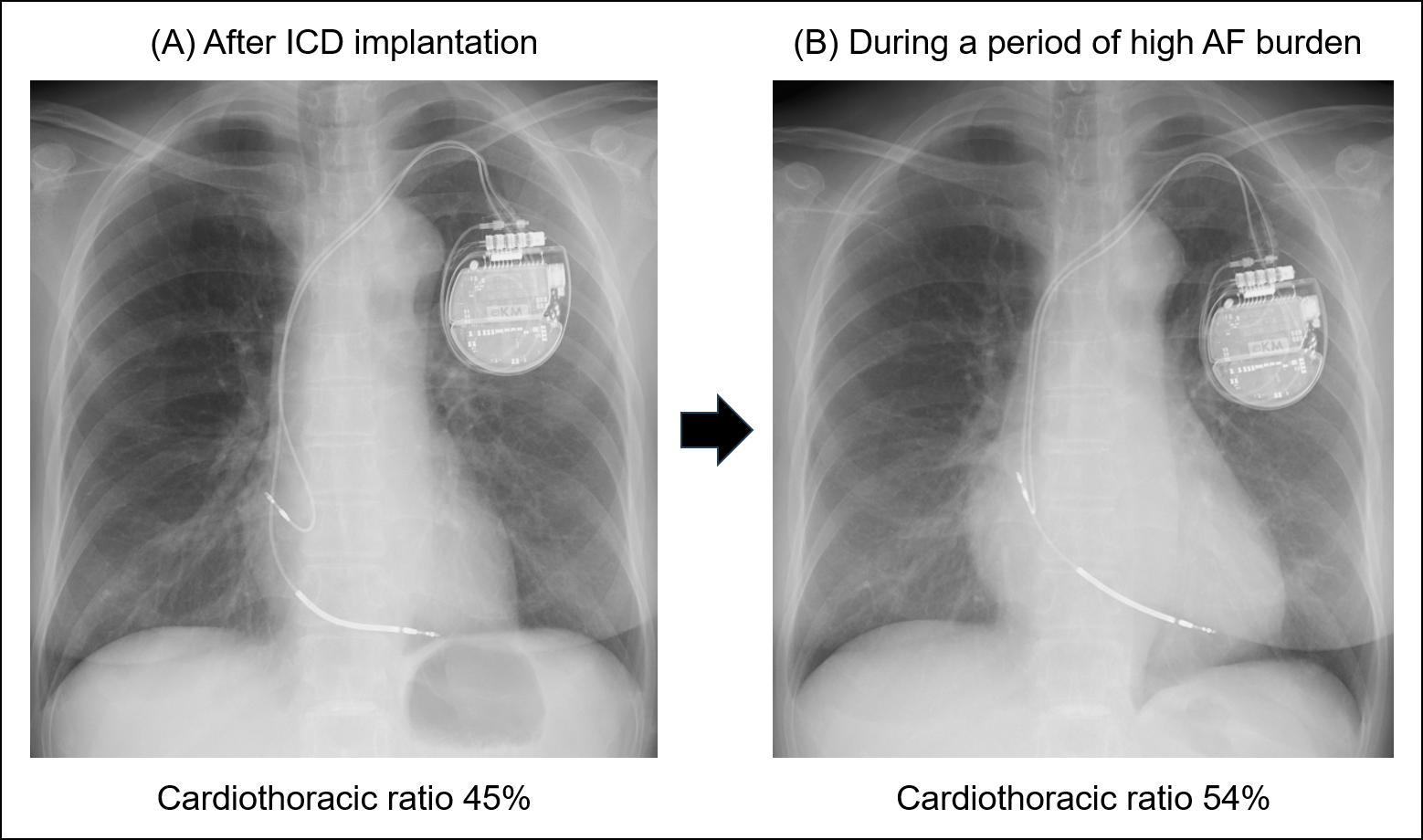

Supplement: Supplementary file 2 — Supplemental Figure 2 Chest radiographs obtained after implantable cardioverter‐defibrillator implantation (A) and during a period of high atrial fibrillation burden (B). (B) Compared with (A), the chest radiograph demonstrates worsening pulmonary congestion, and the cardiothoracic ratio increased from 45% to 54%. AF, atrial fibrillation; ICD, implantable cardioverter‐defibrillator. [file JOA3-42-e70265-s002.tif]

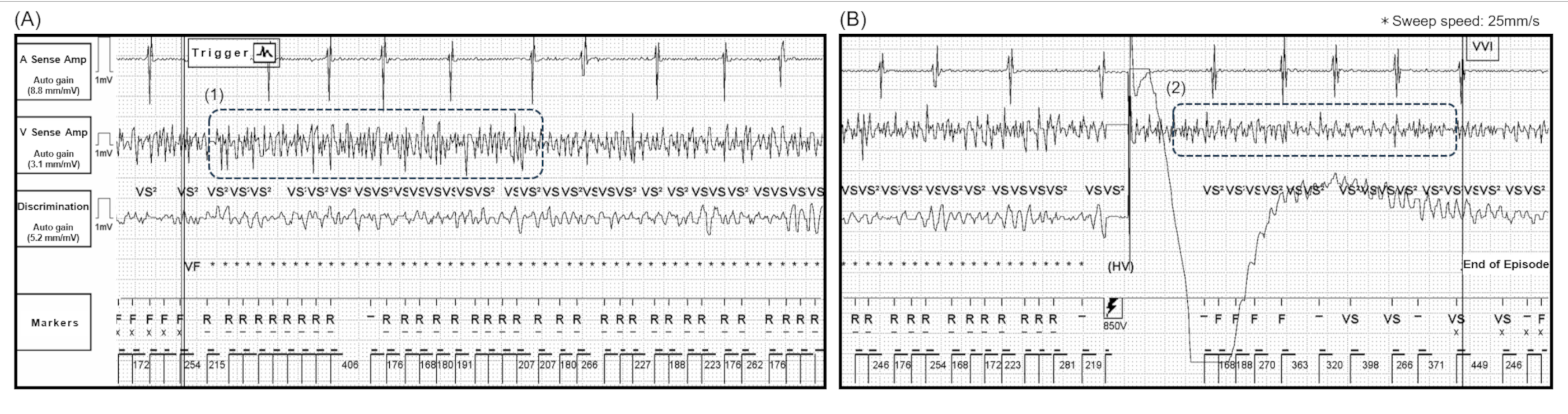

Supplement: Supplementary file 3 — Supplemental Figure 3 The intracardiac electrogram from ventricular fibrillation detection to the delivery of the first shock is shown. When comparing the amplitude of the near‐field right ventricular signal in panel (A‐1) with panel (B‐2), it is evident that the signal amplitude in panel (B‐2) is markedly reduced. This progressive reduction in amplitude resulted in undersensing after the shock, which subsequently triggered ventricular fibrillation therapy assurance activation. F, ventricular interval within ventricular fibrillation zone; HV, high‐voltage shock therapy; R, reconfirmed interval; STIM, stimulus of anti‐tachycardia pacing; T, ventricular interval within ventricular tachycardia zone; VS, Ventricular Sensed event; VS2, low‐amplitude R‐wave signal detected by the coil‐to‐can sensing channel (discrimination channel); VVI, ventricular pacing, ventricular sensing, inhibited response. [file JOA3-42-e70265-s003.tif]
